# Fault2SHA Central Apennines database and structuring active fault data for seismic hazard assessment

**DOI:** 10.1038/s41597-021-00868-0

**Published:** 2021-03-22

**Authors:** Joanna Faure Walker, Paolo Boncio, Bruno Pace, Gerald Roberts, Lucilla Benedetti, Oona Scotti, Francesco Visini, Laura Peruzza

**Affiliations:** 1 UCL IRDR, Institute for Risk and Disaster Reduction University College London, Gower Street, London, WC1E 6BT UK; 2 DiSPUTer Department, Università degli Studi G. d’Annunzio Chieti e Pescara, Chieti, Italy; 3 Department of Earth and Planetary Sciences, Birkbeck College, Malet Street, London, WC1E 7HX UK; 4 Aix-Marseille Université, CEREGE CNRS-IRD UMR 34, Aix en Provence, France; 5 Bureau d’Evaluation des Risques Sismiques pour la Sûreté des Installations, IRSN, Fontenay-aux-Roses, France; 6 INGV - Istituto Nazionale di Geofisica e Vulcanologia, Pisa, Italy; 7 National Institute of Oceanography and Applied Geophysics - OGS, Borgo Grotta Gigante 42/C, 34010 Sgonico (Trieste), Italy

**Keywords:** Tectonics, Natural hazards

## Abstract

We present a database of field data for active faults in the central Apennines, Italy, including trace, fault and main fault locations with activity and location certainties, and slip-rate, slip-vector and surface geometry data. As advances occur in our capability to create more detailed fault-based hazard models, depending on the availability of primary data and observations, it is desirable that such data can be organized in a way that is easily understood and incorporated into present and future models. The database structure presented herein aims to assist this process. We recommend stating what observations have led to different location and activity certainty and presenting slip-rate data with point location coordinates of where the data were collected with the time periods over which they were calculated. Such data reporting allows more complete uncertainty analyses in hazard and risk modelling. The data and maps are available as kmz, kml, and geopackage files with the data presented in spreadsheet files and the map coordinates as txt files. The files are available at: 10.1594/PANGAEA.922582.

## Abstract (summary)

**Table:** 

Measurement(s)	active geological fault
Technology Type(s)	digital curation
Factor Type(s)	location
Sample Characteristic - Environment	geological fault
Sample Characteristic - Location	Central Apennines

Machine-accessible metadata file describing the reported data: 10.6084/m9.figshare.14102021

## Background & Summary

Presenting a new conceptual framework to gather fault data useful for SHA (seismic hazard assessment) was identified as a key challenge by the Fault2SHA Working Group (http://fault2sha.net/)^1^, established within the European Seismological Commission (ESC) in 2016. To improve fault-based SHA, field geologists should provide the relevant observations, analysts should interpret field data appropriately^2^, and the full range of uncertainties associated with the characterization of faults should be correctly understood and propagated in computations^3^. To overcome the barriers that exist for the above, due to the different experience and expertise of participants, we, the Fault2SHA Central Apennines Laboratory, have brought together representatives from research groups across multiple institutions comprising field geologists, seismic hazard modellers and practitioners to create the Fault2SHA Central Apennines Database^4^.

Fault geometries and associated slip-rates are critical in determining earthquake locations and occurrence rates^2,3,5^ in SHA: we acknowledge that including these data is key to improving earthquake hazard and risk estimates. Currently, most hazard models rely principally on historical earthquake rates, but these may not be representative of longer term rates (either overestimating or underestimating as typical recurrence intervals may be hundreds to many thousands of years yet historical records are rarely complete for more than a few hundred years), resulting in a bias in the probability and/or the potential magnitude of earthquakes (e.g. 2011 Great East Japan Earthquake, 2010 Haiti Earthquake^3^). For example, in the central Italian Apennines, where average recurrence intervals on individual faults range from a few hundred to several thousand years^6–9^, there are discrepancies in strain-rates calculated within areas of approximately 2,000 km^2^ using measurements of 15 ± 3kyr long-term multi-seismic-cycle fault offset data and strain-rates calculated using a 700-year-long historical earthquake record (magnitude of completeness, Mc ≥ Mw5.6 since 1349^10^)^11^. Such discrepancies arise because relying on historical earthquake records alone will omit contributions from faults capable of hosting large earthquakes that have not ruptured during the historical record. An enlightening example is the Mt Vettore Fault which hosted the 2016 central Apennines earthquakes that killed three hundred people^12,13–18^; this fault had not ruptured within the historical record, but prior to 2016 it was a mapped fault with palaeoseismic evidence of rupture^19,20^.

In recognition of the need to include long-term slip-rate data, fault-based hazard models have been developed (California^21^, Italy^22^, Greece^23^). Proprietary and open-access tools have been created to help researchers with such endeavours^24–29^. The most commonly used tools infer maximum magnitudes of earthquakes on individual faults from empirical relationships e.g.^30^ and use fault slip-rates to determine average earthquake recurrence rates.

These fault-based hazard models are progressing to include more detail and complexity^29^. The Mw7.8 Kaikoura, New Zealand, earthquake^31^ highlighted that complex rupture scenarios occur including partial and multi-fault ruptures. Variable fault geometry has been shown to influence ground-shaking intensities^32^. Slip-rates, identified as a key source of uncertainties in earthquake probability calculations^21,33^, both coseismic and long-term, can vary significantly along the length of a fault^15,32,34^; utilising detailed multi-point slip-rate data (rather than a single value) can change calculated earthquake rates beyond that expected by intrinsic natural variability^32,34^. Efforts at improving in the provision of data are being made e.g.^35^; however, current seismic source datasets (e.g. https://www.seismofaults.eu/,^36^) generally do not provide sufficient detail in mapped traces and slip-rate data to include the above, and do not give guidelines on how to aggregate mapped structures into a seismic source; this limits the interrogation of alternative rupture scenarios and the inclusion of detailed fault geometry and slip-rate profiles.

**Fig. 1:**
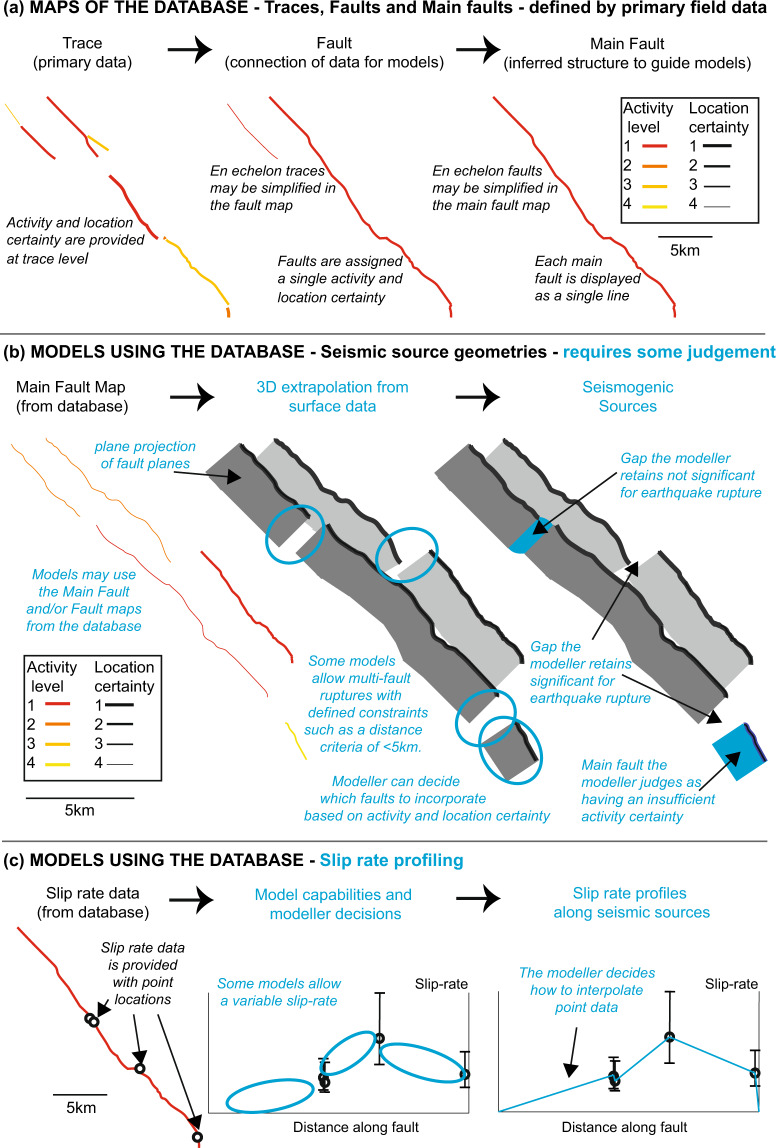
From primary data to modeller decision making. (**a**) An example showing the relationship between traces, faults and main faults. In the trace, fault and main fault maps, the thicker the line, the higher the location certainty and the colour shows the activity certainty (red, dark orange, light orange, yellow show levels 1, 2, 3 and 4 respectively) – see Tables 1 and 2 for details. (**b**) An example of how a modeller may turn main faults in the database into seismic sources using a multi-fault rupture model and a minimum activity certainty level criterium for inclusion; in this example, the maximum extent of ruptures across multiple faults is determined using a distance between faults criteria. Blue text signifies modelling decisions, black text represents information directly from the database. Locations affected by such modeller decisions are shown with blue ovals with the parts of the faults affected shown in blue. Note a modeller can alternatively choose to use the fault map to create alternate rupture scenarios. (**c**) An example of how a modeller may interpolate the point level slip-rate data for inclusion in a fault-based seismic hazard model.

Herein, we present a recommended database format with trace, fault and main fault maps using the central Italian Apennines as our prototypal area. The data can be easily incorporated into fault-based SHA calculations^37^, consistent with FAIR principles^38^, and the schema can be adapted for other regions. Included slip-rate data should have published coordinates and time caps. The advantages this database brings are that (1) trace-level data shows local map confidence, (2) the fault map allows modellers to interrogate different seismic rupture scenarios involving partial or multi fault ruptures, (3) the main fault map guides towards seismic sources, and (4) the provision of point data allows freedom for different interpolations of slip-rates and geometry (Fig. 1).

## Methods

**Fig. 2:**
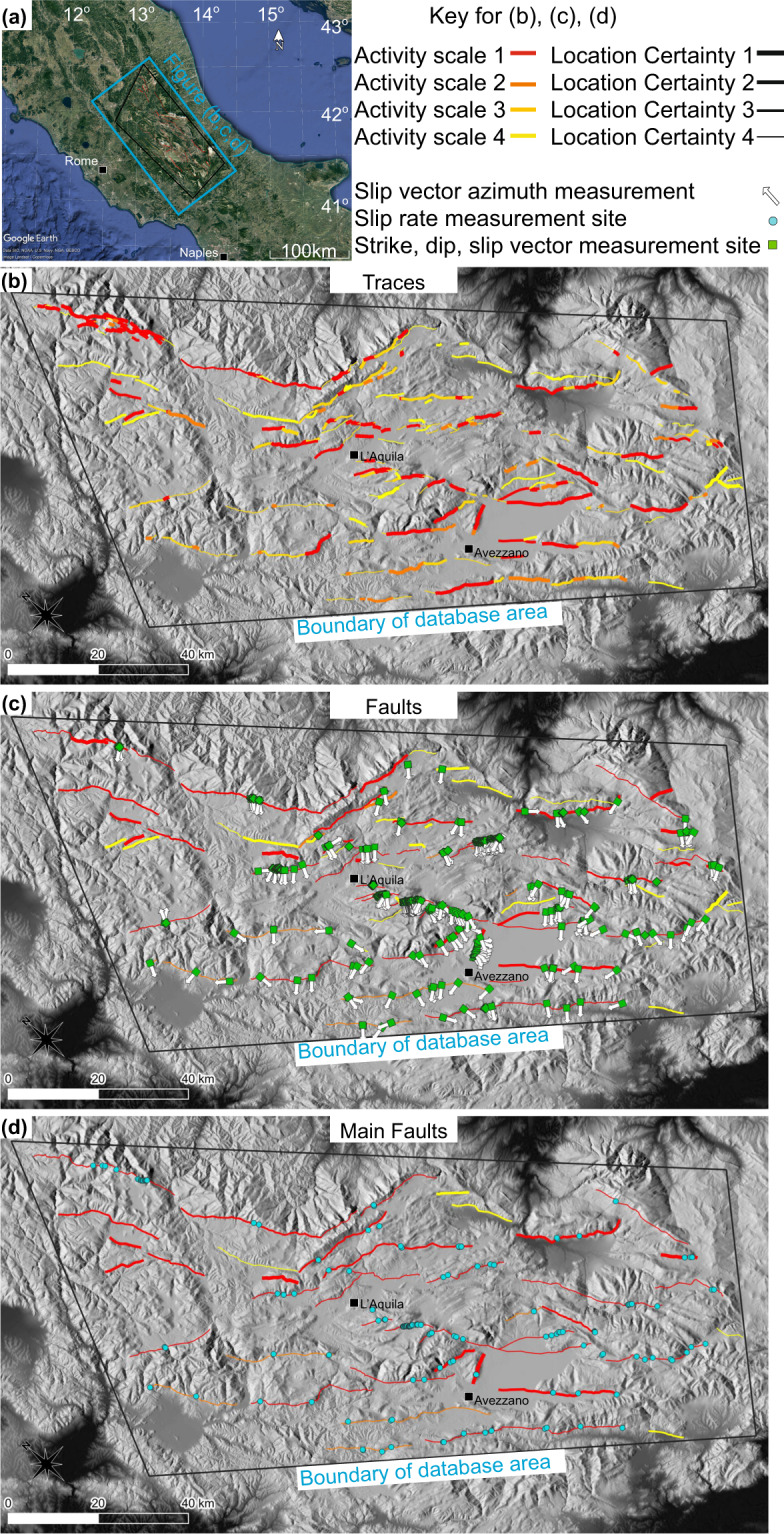
Location maps showing the traces, faults and main faults in the database. (**a**) Location map, (**b,c,d**) maps of the geographical area covered by the database showing the individual traces (**b**), faults (**c**), and main faults (**d**). The MainFaultOption choices shown are A1, B1 and C1 (see MainFaultSelection table for explanation). The thickness of the lines signifies the certainty in the trace location (thickest = most certain, see “traceLocationScale” for detailed explanation). The colours of the lines signify the certainty in there being earthquake activity during the Late Pleistocene – Holocene (hierarchy of highest to lowest certainty is red, dark orange, light orange, yellow) see “traceActivityScale”. Faults are drawn with the lowest traceLocationScale and highest traceActivityScale of the traces they comprise. Main faults are drawn with the lowest faultLocationScale and highest faultActivityScale of the faults they comprise. The sites of local surface strike, dip and or slip vector azimuth and plunge within the LocalGeometryKinematics table are shown in (**c**) as a green diamond with measured slip vector azimuths recorded in the database shown as arrows. The locations of slip-rate and/or throw-rate measurements from the SlipRate table are shown in (**d**) as blue circles. Imagery is from GoogleEarth and DEM from TINITALY^102^.

**Fig. 3:**
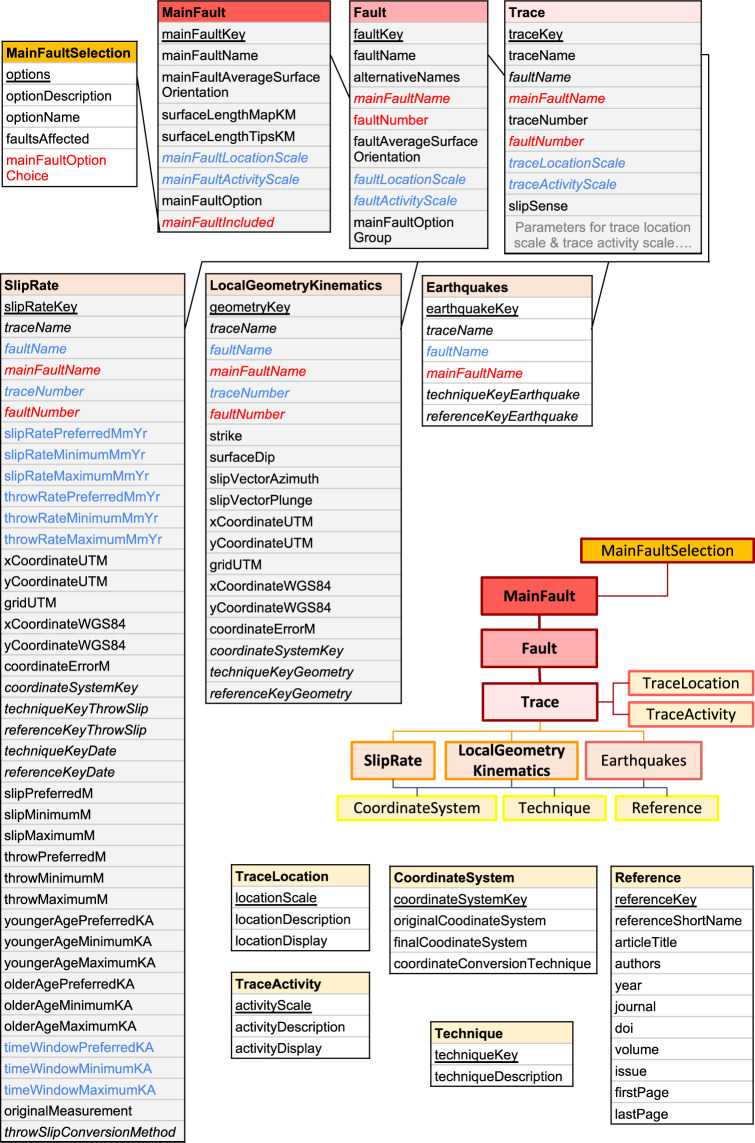
Database schema showing how different tables relate to each other and the fields within the tables. The unique identifier for each table is underlined. Fields that connect to other tables are in italics. Derived quantities calculated from other fields are shown in blue. Field headings shown in red represent those that change dependent on the main fault option selected. Tables shaded grey have map layers associated with them.

The database provides separate maps at the trace, fault and main fault scale (see following sections for definitions and Fig. 2 for the maps) and provides tables with associated properties for these three resolutions of mapping (see Fig. 3 for a list of fields in each table). The database further provides point level data for local geometry and slip rates including the techniques used for their measurement, the original coordinate systems used for the locations, and the reference from which the data was obtained (Fig. 3). The maps and accompanying data have been assembled from the literature, with some new work contributing to the maps. We aim to include all faults in the region with evidence of Late Pleistocene - Holocene activity.

### Trace, Fault and Main Fault Maps

In the central Apennines database, we have defined 43 or 44 individual main faults (depending on main fault selection chosen, selection is made from a choice of 49 main faults, but only 43 or 44 are independent) made up of 87 faults which are discretised into 312 traces.

#### Traces

Traces report data at the primary level, with the location certainty and activity scale being defined at this level. The extent of a trace is determined by the distance over which the criteria for determining location certainty and activity remain the same. There are no length restrictions for trace-level mapping. The trace-level mapping is envisioned to be of interest to those collecting primary data in the field including those wanting to undertake high-resolution structural studies. Trace-level mapping is of interest for fault displacement hazard, where the detail of fault mapping is of primary importance. This detail of mapping allows end-users to identify what evidence has been used to define the trace location certainty and activity certainty at this scale and hence how the lower resolution mapping location and activity certainties have been determined.

The trace map and table data have been created using a combination of published papers including capable fault maps^6,8,11,15,17,22,32,39–44^, (http://sgi2.isprambiente.it/ithacaweb/Mappatura.asp), published geological maps^45–55^, (https://www.isprambiente.gov.it/Media/carg/index.html) and further publications including palaeoseismological trench site data, macroseismic earthquake data, geomorphological and geological observations, seismic profiling, air photographs, LiDAR and InSAR^7–9,15,16,19,56–97^, new fieldwork, and new DEM and aerial photography interpretation.

The traces are displayed according to their location certainty scale (how precisely their location is known conveyed by varying line thickness) and activity scale (the degree of evidence for tectonic displacement along the trace during the Holocene-Late Pleistocene conveyed via different coloured lines); see Tables 1 and 2 for observations leading to the location and activity scales for individual traces. Each scale has four levels, with 1 denoting the highest confidence and 4 the lowest. The “Trace” table includes the trace attributes so that the calculation of location and activity certainty can be traced back to the primary observations that describe this in the database.

**Table 1:** Trace location scale adopted in the database.

Trace Location Scale	Line Thickness Display (pixels)	Location Description	Certainty Calculation
1	8	Location Certain The trace is mapped to within a few metres at the surface	At least one of the following must have been directly observed (i.e. ground-truthed): a bedrock fault scarp, a sharp Quaternary scarp, real-time earthquake surface ruptures.
2	6	Approximate location certain, but local alterations possible The trace is known within tens of metres The trace may be precisely known at specific points, but requires interpolation between these.	At least one of the following must have been observed: a bedrock fault scarp or sharp Quaternary scarp through remote sensing, palaeoseismic trenching with evidence of previous earthquakes, or offset Late Pleistocene deposits measured via topographic offsets. This certainty level is also assigned if there is a change in slope AND incised drainage AND along another trace belonging to the same fault there are direct observations evidence pertaining to certainty scale 1.
3	4	Location uncertain, but with some local constraints The trace follows the approximate path shown, but new evidence could allow for significant alterations	At least one of the following has been observed: incised drainage, geophysical data showing evidence of faulting, or earthquake displacements from InSAR or other remote sensing
4	2	Location uncertain Future evidence could allow significant changes to the fault trace location	There are not sufficient observations for levels 1, 2 or 3

**Table 2:** Trace activity scale adopted in the database.

Trace Activity Scale	Activity Display	Activity Description	Activity Calculation
**1**	Red	Dated displacement during Late Pleistocene - Holocene	At least one of the following must have been observed: ground-truthed real-time earthquake surface ruptures, earthquake displacements from InSAR or other remote sensing, a palaeoseismic trench with evidence of tectonic offsets that have date constraints, or cosmogenic dating providing evidence of tectonic offsets within the Late Pleistocene - Holocene.
**2**	Dark Orange	Evidence of Late Pleistocene - Holocene displacement, but without *in situ* dated Late Pleistocene Holocene displacement	Local displacement constrained to be within the Late Pleistocene – Holocene through regional dating constraints: a scarp profile showing offsets features that have regional dating constraints (Late Pleistocene - Holocene age constraints) but that have not been dated specifically along the trace (scarp profiles that offset Late Pleistocene-Holocene).
**3**	Light Orange	Geologic (displaced Middle Pleistocene deposits) or geomorphic evidence of potential fault activity, but this has not been confirmed as Late Pleistocene. The trace is on the same fault as a trace with activity (1) or (2).	Criteria needed for activity (1) or (2) are present for a different trace belonging to the same fault, but not present on this trace.
**4**	Yellow	Geologic (displaced Middle Pleistocene deposits) or geomorphic evidence of potential fault activity, but this has not been confirmed as Late Pleistocene	There are not sufficient observations for levels 1, 2 or 3.

The “trace location” certainty is greatest for level 1 location where the fault trace is certain within a few metres at the surface and confirmed during field investigations evidencing Late Pleistocene - Holocene displacement. Level 2 location represents traces known within a few or tens of metres, so local uncertainty in the mapped position of the trace is possible; the trace may be constrained precisely at particular points (e.g. by palaeoseismic trenches or geophysical observations) but not continuously and thus some parts require local interpolation. Level 3 location traces have location uncertainty, but have some local constraints such that the trace follows the approximate path shown with evidence for a local fault but not locating the trace precisely (i.e. not continuously within a few or tens of metres), new evidence could allow for significant alterations. Level 4 represents the lowest level of certainty in location, the location is uncertain by tens of metres or more and there could be significant alterations in future versions of the fault map. Table 1 provides the full criteria for certainty levels at each location. All traces included in the map have some geologic or geomorphic evidence of activity, however not all have this constrained to the Late Pleistocene - Holocene with certainty (see Table 1 for details).

The “trace activity” criteria have been inspired by the classification schemes in previous works^39^, however we have identified specific requirements among criteria listed within the database so that users can see where the scale values have come from and what observations have been made along each trace (see Table 2). Level 1 represents traces that have displacement during the Late Pleistocene – Holocene that has been dated at points along the trace. There must be dated evidence of tectonic surface ruptures during historical or contemporary rupture. Therefore, at least one of the following must have been observed for a trace to be assigned activity level 1: ground-truthed real-time earthquake surface ruptures, earthquake displacements from InSAR or other remote sensing, a palaeoseismic trench with evidence of tectonic offsets that have date constraints (this can include palaeoseismic trench site dating techniques that rely on *in situ* samples that are geochemical matches, for example tephrachronology), or cosmogenic dating providing evidence of tectonic offsets within the Late Pleistocene - Holocene. Level 2 activity has evidence of Late Pleistocene displacement with dates inferred from regional level dating, but without *in situ* dated Late Pleistocene - Holocene displacement. For example, a scarp profile showing offsets features that have regional dating constraints. Level 3 activity for a trace is assigned where there is geologic (displaced Middle Pleistocene deposits) or geomorphic evidence of potential fault activity, but this has not been confirmed as Late Pleistocene - Holocene, this can represent traces where the trace is on the same fault as a trace with activity 1or 2, but itself does not meet the criteria for activity level 1 or 2. Level 4 activity traces have geologic (displaced Middle Pleistocene deposits) or geomorphic evidence of potential fault activity, but this has not been confirmed as Late Pleistocene - Holocene.

#### Faults

Faults display how the traces are connected at the surface and/or at depth. How the traces connect to form faults is based on fault geometry continuation (two traces can start and finish within a few metres from each other or may be kilometres apart), continuation of Late Pleistocene-Holocene offset and total offset across the fault and known earthquake ruptures. In showing how traces are connected, the fault map is of use to those wanting to understand the structures of the region.

All traces belong to one of the faults in the database. However, each fault is represented by a single, continuous map line in the “Fault” map, therefore what are interpreted as secondary traces parallel, quasi parallel or branching to the main fault rather than forming a continuation of the fault, interpreted as a branch, splay or other synthetic or antithetic structures do not appear at the “fault” level (see Figs. 1a, 2c,d). Traces may overlap in an en echelon form, in these cases either one or more of the overlapping traces will be excluded from the fault map and/or adapted so that a single line can be drawn. The single lines allow the faults to be more easily incorporated into models. Within the database, faults have a minimum length of 1 km. Note that faults can contain traces with different levels of location and activity certainty. Faults are assigned the highest level of activity among their constituent traces, while they have the lowest level of location certainty from among the constituent traces. This is because if activity can be confirmed along a fault, it is assumed the fault has that activity level and other traces either have not been studied sufficiently or the evidence is not apparent. All traces belonging to a fault contribute to the fault activity score. For the location certainty, only the traces contributing directly to the mapped line are considered (so as to avoid decreasing the certainty due to less well constrained splays, synthetic and antithetic structures).

#### Main faults

Main faults represent how faults have been interpreted to be linked at depth. Connection of one or more faults into a single main fault has been inferred using factors such as continuation of surface geometry (fewer constraints than for faults), total offsets across the faults with the total offsets decreasing to zero at main fault tips, surface slip vectors convergence, and laterally-continuous rupture during contemporary, historical and palaeo earthquakes. We recommend that this scale of mapping is useful for input into current seismic hazard models.

In some cases more than one main fault is known to have ruptured in a single earthquake, in such cases, multiple faults across more than one main fault may represent a rupture, such as during the 1915 M 7.0 earthquake which ruptured the Fucino Ovindoli Pezza, Magnola (or Fucino Magnola), Cerchio Pescina Parasano, and Trasacco main faults comprising (among others), the San Benedetto Dei Marsi, Marsicana Highway, Parasano, Magnola and Trasacco and Luco Dei Marsi faults^57,60,69,96^. In those cases, the identification and naming of faults and main faults is guided by both geological and historical literature reasons, in the sense that some faults are left separated as defined and named in the literature, even though they have ruptured contemporaneously in past earthquakes. Therefore, there is no one-to-one relation between faults or main faults and “earthquake segments” within which ruptures are expected to be confined. To allow for different interpretations of seismic sources, modellers may wish to utilise either the main faults (Fig. 2d) or the faults (Fig. 2c). Each main fault is represented by a single, continuous map line in the “main faults” map, therefore the geographic representation of the main fault on the map may differ locally from its constituent faults as some detail is necessarily simplified if there are, for example, en echelon faults, and main faults may comprise synthetic or antithetic faults, but only the main fault line will be displayed at the main fault level map (see Figs. 1a, 2b,c). Within the database, main faults have a minimum length of 5 km. Note that main faults can contain faults with different levels of location and activity certainty. Main faults are assigned the highest level of activity among their constituent faults. Main faults have the lowest level of location certainty from among the constituent faults which form the mapped main fault (excluding synthetic and antithetic faults).

#### Further considerations for the maps

We recognise that there may be different interpretations of how faults join to form main faults. Therefore, we have provided three sets of options for particular main faults in order to allow different interpretations to be considered in hazard modelling. For example, the first concerns how the northern end of the San Benedetto Dei Marsi fault continues near Celano. Total fault offsets suggest that the San Benedetto Dei Marsi fault likely joins the Ovindoli Pezza to form a single main fault (herein named Fucino Ovindoli Pezza) as otherwise there would be very sharp decreases in total throw towards Celano^43^. However, there is no known direct geologic or geomorphic evidence of continuation of the Ovindoli Pezza fault through Celano town and the Celano paleolandslide or across the Miocene rocks and Lower-Middle Pleistocene continental units around Celano. In addition, interpretation of seismic reflection data could indicate that the total geologic displacement along the San Benedetto Dei Marsi fault goes to zero at Celano^85^. Therefore, considering the ruptures during the 1915 Fucino earthquake, we have also provided the option of the San Benedetto Dei Marsi Fault joining the Magnola Fault to form the Fucino Magnola main fault. The second option concerns whether the Frattura and Castel Di Ieri faults connect to form the Scanno main fault or not. The third option concerns connection of faults within the Upper Aterno Valley, specifically whether the Mt Stabiata fault connects to the Paganica and San Demetrio Ne Vestini faults or to the San Pio Delle Camere fault. We suggest that any hazard calculations must consider the alternate hypotheses as there is not a consensus for these main faults.

Providing the three map levels allows users to interrogate how the fault and main fault maps have been built up from the trace level observations. The trace map shows where observations have been made, to what levels of certainty have been achieved, and where gaps in knowledge exist. This differs from the approach of the existing seismogenic fault mapping for the region. ITHACA (ITaly Hazards from CApable faulting) is an Italian database produced by ISPRA (Istituto Superiore per la Protezione e la Ricerca Ambientale) that provides detailed fault traces for capable faults in Italy and surrounding areas (http://sgi2.isprambiente.it/ithacaweb/Mappatura.asp). The ITHACA map aims to include all faults referred to in the literature and provides geological information at a high spatial resolution; however, it does not display how the mapped structures connect and the high detail – though a highly valuable resource for local study and geological investigations – may be beyond current seismic hazard model capability. Conversely, DISS (Database of Individual Seismogenic Sources)^98^ provides a lower resolution map, which aims to give both single-rupture (individual) or multi-rupture (composite) seismic sources (within DISS a seismogenic source is an existing or hypothesized fault capable of producing magnitude Mw 5.5 and larger earthquakes, but a seismogenic source does not necessarily correspond/coincide with real fault field data). To accomplish the complexities and divergency in data interpretations (shown to be needed by the L’Aquila 2009 and Amatrice 2016 earthquake sequences) DISS now provides a table of “debated” seismogenic sources which comprise faults within the literature for which the authors of DISS do not consider have sufficient evidence, or are “reliable enough” for inclusion in the main database. Despite this, the “seismic sources” in the DISS are inferred simple structures and do not show how faults (and higher resolution traces) have been mapped in the field. The resolution of the data is not at a scale that we advocate as being needed for improving seismic hazard assessments, especially for the case of high-resolution calculations required for critical infrastructure, and it does not allow for modellers to build alternate rupture scenarios through consideration of higher resolution geological structures.

### Point data: slip rate, local surface geometry, and kinematics

Slip rate, local surface geometry and kinematic data were extracted from existing publications^9,11,13,17,18,32,40–42,68,80,99,100^. The database does not aim to include all data that has been published; for data to be included, it must: (1) have been published in a peer-reviewed journal prior to the publication of the database; (2) have been provided with sufficient location precision for point data location to be recorded (if the coordinates were not published (for example a publication may only show a map with points marked), but the original authors have locations recorded, this data can be included in the database; an attempt was made to contact authors for inclusion of such data, but a lack of recorded coordinates prevailed); (3) pass quality control (for example data with better constraints is available for the same location, the less well-constrained data are considered redundant); and (4) slip-rate or throw-rate measurements, either published directly or as slip or throw measurements, must have the time period for the calculation recorded. We hope the database will encourage future data to be published with location coordinates so it can be included. The authors of the database recognise that there may be data in the literature that was missed, but ought to have been included, in compiling the database. If readers have data to contribute to the database, we ask that they fill in the “Fault2SHA_CentralAPennines_Database_NewDataForm_2021_v1” and send it to the corresponding author via email with title “Fault2SHA Central Apennines Database New Data Form”. In the form the columns are editable where not reliant on other columns for information, but cells shown in yellow should not be edited. The user will be contacted if further information is required.

We compared the data included from different sources to check whether there was consistency. We found consistency between, for example, slip-rates measured using different techniques which adds to the robustness of the data. For example, along the PianoDiCampoFelice trace of the CampoFelice fault, the Forme trace along the Magnola fault, the ParasanoEast trace along the Parasano fault, the SanSebastianoOrtonaDeiMarsi trace along the San Sebastiano fault, the TrasaccoCollelongo trace along the Trasacco fault, the TreMontiPaterno trace along the Tre Monti fault, and the Fiamignano trace along the ValleDelSalto fault, the slip-rate data in the database measured from topographic offsets are consistent with that reported using cosmogenic dating of the fault scarp^8^.

#### Methods used within the database for values within the point data tables

The original values within the SlipRate and LocalGeometryKinematics tables come from published sources. Within the database, the method used to collect the original data is cited and we direct readers interested in particular values to the original sources. For each method listed in the “Technique” table we provide a short summary. We also state where data have been converted and how – for example slip may be derived from throw data using a nearby measurement of fault dip or plunge as stated.

### Creation of gpkg, kmz and xlxs files

The data tables were compiled using Google Sheets and Microsoft Excel and then extracted as individual csv files. The maps were drawn in GoogleEarth. The map kml files were extracted for use within QGIS, where they were joined to the corresponding data tables. The map lines were assigned colours and thicknesses based on the values in the attributes tables for the activity and location certainty. The geopackage and kml files were then extracted from QGIS and the final kmz was saved within GoogleEarth.

## Data Records

### Database availability

The database including the maps and attribute tables and the data extraction codes for example SHA codes are available at 10.1594/PANGAEA.922582^4^ in the following formats:

Database:

1. Zipped keyhole markup language (Fault2SHA_CentralApennines_Database_2021_v1.kmz and Fault2SHA_CentralApennines_Database_2021_v1.kml) file for use in GoogleEarth – these currently display map lines of the main faults, faults, and traces each accompanied by metadata and point locations of slip rate and local geometry and kinematics accompanied by the metadata. The “MainFault” folder contains the individual main fault lines showing how the faults have been interpreted to be connected at depth. The MainFault Option Groups appear as folders and allow one option within each group to be selected to avoid double mapping of any fault. Any fields in the tables within the kml and kmz files which are dependent on the MainFaultOptionChoice have the different possibilities listed within the tables with the options they belong to stated in brackets. The “Fault” folder shows how the traces have been interpreted to be connected. The “Trace” folder comprises the individual traces. See Tables 1 and 2 for the key to the colours and line thickness. The “Trace”, “Fault” and “MainFault” Table fields associated with each trace, fault and main fault can be accessed by clicking on the trace, fault and main fault name respectively within the folders or clicking on the relevant line on the map. The “SlipRate” and “LocalGeometryKinematics” folders contain map points for each measurement in the database, named according to the trace that the measurement belongs to. The fields from the tables can be accessed through clicking the entry within the list in the folder or the relevant point on the map. We have provided alternate MainFault selection options in the shapefiles. Therefore, only one option from each option group should be chosen when displaying a map at the Main Fault level. This file has been tested in GoogleEarth Pro 7.3.3.7699
2. Geopackage (Fault2SHA_CentralApennines_Database_2021_v1.gpkg) file compatible with open access GIS programmes containing both the maps and attribute tables for the data in the database. The “MainFault”, “Fault” and “Trace” layers contain both the maps of the main faults, faults and traces respectively and their attributes tables. The “SlipRate” and “LocalGeometryKinematics” layers contain both the point locations for the relevant measurements and their attributes tables. The remaining tables (TraceLocation, TraceActivity, Earthquakes, CoordinateSystem, Technique, Reference) appear as attribute tables as no geometry layers. Within the gpkg file, the user will need to select which main faults to display is the MainFault layer to avoid duplication of faults (the MainFault layer contains all the possible main faults). For example, within QGIS 3.8 this can be achieved through selecting the MainFault layer, opening Layer – Layer Properties – Source “Query Builder” and using a query to select the desired MainFaults, for example to select option 1 within each group use the query: “mainFaultOption” = 0 OR “mainFaultOption” = ”A1” OR “mainFaultOption” = ”B1” OR “mainFaultOption” = ”C1”. Any fields in other tables which are dependent on the MainFaultOptionChoice have the different possibilities listed within the tables with the options they belong to stated in brackets. This file has been tested in QGIS 3.8.
3. An excel spreadsheet (Fault2SHA_CentralApennines_Database_2021_v1.xlsb and Fault2SHA_CentralApennines_Database_2021_v1.xlxs) of the database attribute tables, with each table on a separate tab. Unlike the kml, kmz and gpkg files, the excel sheet allows dynamic updating of the fields dependent on the MainFaultOptionChoice and thus only the current option is displayed. These files have been tested in Excel 16.35 and Excel 2013 respectively. By default, the worksheets other than “MainFaultSelection” are locked, the password is “Fault2SHA”.
4. Tab delimited text files for the longitude and latitude coordinates of the main fault and fault maps, organised in two zipped folders with each main fault or fault represented by an individual file, with folder names: “Fault2SHA_CentralApennines_Database_MainFaults_lonlat_2021_v1” and “Fault2SHA_CentralApennines_Database_Faults_lonlat_2021_v1”.

Additional files:

1. An excel spreadsheet (“Fault2SHA_CentralApennines_Database_NewDataForm_2021_v1.xlbs” and “Fault2SHA_CentralApennines_Database_NewDataForm_2021_v1.xlsx) in the format of the database but with most fields empty. This file is available to anyone who would like to propose data to be included in future versions of the database.
2. A MATLAB script (data2FiSH_2021_v1.m) for extraction of the data from the database in a ready-to-use format for the FiSH code and an accompanying readme text file (README_script_data2FISH_2021_v1.txt) with instructions for its use. These are contained within the “Data2FiSH_2021_v1” folder along with 2 supporting MATLAB scripts: “utm2deg.m” and “deg2utm.m”.

If the user has compatibility issues with software versions please contact the corresponding author.

### Database tables

Here we provide an overview of the database structure, how the tables are connected and the different fields within each table (Fig. 3).

The unique identifier for each table is underlined. Fields that connect to other tables are in italics. Derived quantities calculated from other fields are shown in blue and fields that are dynamic depending on the Main Fault option selection made are shown in red in the database. The type of data within each column is shown in brackets following the name of the column.

#### MainFaultSelection

The main fault selection table provides the opportunity for the user to select different possible main fault configurations from the dropdown lists. Main fault options are only provided for where there are alternative configurations, i.e. where there is only one configuration given, there is no option in this table.

**options** (String) – a unique identifier for each available option for main faults where there is more than one main fault configuration provided. Each option is split into a lettered group and within the group each option is given a number, such that each option has a string form of XX, where the first digit is a capital letter pertaining to the group of options and the second digit is an integer referring to the options within that group.

**optionDescription** (String) – describes the main fault configuration within the option

**optionName** (String) – provides a descriptive name for the option group, it has the form XXX – X, where “XXX” before the dash represents the name (can be of any length) and the “X” after the dash is the option group letter

**faultsAffected** (String) – lists the different faults that will be affected by the selection made within the option group

**mainFaultOptionChoice** (Dropdown, String) – this is a dropdown list in which one of the options within the group must be selected. The choice selected here will change the dynamic fields in other tables that are affected by the main fault selection.

#### MainFault

The main fault table provides a list of the main faults included in the database together with their strike and main fault-level activity and location certainty scale. Main faults comprise one or more faults. It further shows which main faults are independent of main fault options and which will only be present under particular main fault option selections (see MainFaultSelection table).

**mainFaultKey** (Integer) – a unique identifier for each main fault

**mainFaultName** (String) – a unique identifier for each main fault, the name assigned to each main fault in the database, provided in alphabetical order. Names were chosen including consideration of prevalence in the literature and geographic or political features to help identify them.

**mainFaultAverageSurfaceOrientation** (Integer) – an approximate measure of the strike of the main fault, measured in degrees from north, taken as the angle between the fault tip locations at the surface. Although this value gives an overall gauge of the orientation of the fault, we note that the geometry of individual traces is preferable in modelling and that the fault average strike has uncertainties due to uncertainties regarding the locations of fault tips.

**surfaceLengthMapKM** (Integer) – Length in km of the main fault measured as the length of the detailed mapped line at the surface (sum of the distances between each coordinate point) along the main main fault (i.e. it does not consider branches, splays, synthetic or antithetic structures etc.)

**surfaceLengthTipsKM** (Integer) – Length in km of the main fault measured as the length of the line drawn between the two surface tips of the main fault (i.e. it does not consider branches, splays, synthetic or antithetic structures etc.) The surfaceLengthTipsKM will be less than or equal to the surfaceLengthMapKM.

***mainFaultLocationScale*** (Integer) – see TraceLocation table. This provides a gauge on how certain the location of the main fault is. The score is derived from the sections that comprise the fault and is the lowest level of location certainty of the faults that form the main fault (excluding antithetic and synthetic structures not along the main main fault line), in turn the scores of the faults are derived from the trace scores that comprise the fault (excluding antithetic and synthetic structures not along the fault line).

***mainFaultActivityScale*** (Integer) – see TraceActivity table. This provides a gauge on how certain the activity of main fault is during the Late Pleistocene-Holocene. The score is derived from the scores of the faults that comprise the main fault (including synthetic and antithetic structures), being the highest level of activity of the faults that form the main fault, in turn the activity score of the faults are derived from the traces that comprise them.

**mainFaultOption** (String) – shows whether and which options apply to the main fault. If there is only one option for the main fault, then this field is 0. For main faults which are affected by the main fault option choice, the main fault option which includes the main fault is listed.

***mainFaultIncluded*** (Integer) – This is a dynamic field that shows whether the main fault is included in the current main fault options selected (1) or not (0). Note this field is not included within the kml, kmz or gpkg files.

#### Fault

The fault table provides a list of the faults included in the database and the main faults they have been assigned to. Faults within a main fault can be across strike or along strike from each other. Faults can comprise one or multiple traces.

**faultKey** (Integer) – a unique identifier for each fault

**faultName** (String) – a unique identifier for each fault, the name assigned to each fault in the database, provided in the order of the faultName and faultSectionNumber (see below). Names were chosen including consideration of prevalence in the literature and geographic or political features to help identify them. Some main faults comprise only one fault and hence share the same properties, in these cases the main fault and fault will share a name.

**alternativeNames** (String) – as some faults are referred to (either entirely or in part) by different names within the literature, here we provide a list of common alternative names used to describe the fault. Note that the alternative names may refer to different faults, main faults or traces by different authors too. This list is not exhaustive, but we hope it captures the majority of references to the faults and thus helps readers by identifying where fault names may have changed and to help them when searching for further information on the desired faults.

***mainFaultName*** (String) – this is a dynamic field, see explanation under Main Fault table. Each fault is assigned to a main fault.

**faultNumber** (String) – this is a dynamic field, each fault is provided with a fault number showing its position along the main fault it has been assigned to. If there is only one fault for a main fault, then the fault number will be 1. Where a main fault has been interpreted as comprising multiple faults, the faults are ordered sequentially from one end to the other (for the central Apennines database this is generally ordered from northwest to southeast). Note there are some examples of main faults where not every fault is considered to be part of the main fault line shown at the surface, for example, the faults may be across strike from each other arranged as synthetic or antithetic structures to the main fault at the surface; in such cases the faults are assigned a number in the hundreds to differentiate them from the faults aligned along strike from each other. Note that for faults along strike from each other the main fault line may not simply be the individual sections amalgamated together so as to allow a single line representation of the fault, for example en echelon structures may be simplified.

**faultAverageSurfaceOrientation** (Integer) – an approximate measure of the strike of the fault, measured in degrees from north, taken as the angle between the fault tip locations at the surface. Although this value gives an overall gauge of the orientation of the fault, we note that the geometry of individual traces is preferable in modelling and that the tip-to-tip strike of the fault has uncertainty due to uncertainties regarding the locations of the fault tips.

***faultLocationScale*** (Integer) – see TraceLocation table. This provides a gauge on how certain the location of the fault is. The score is derived from the observations in the Trace table, being the lowest level of certainty of the traces that form the fault (excluding branches, splays, synthetic or antithetic structures).

***faultActivityScale*** (Integer) – see TraceActivity table. This provides a gauge on how certain the activity of fault is during the Late Pleistocene-Holocene. The score is derived from the observations in the Trace table, being the highest level of activity of the traces that form the fault (including branches or splays, synthetic or antithetic structures).

**mainFaultOptionGroup** (String) – this field shows whether the main fault a fault belongs to is affected by a main fault option. If there is a one to one or many to one mapping from the fault to the main fault then the field appears as “NULL”. However, if there is a many to one or many to many, i.e. that the main fault the fault belongs to can change depending on the main fault option selected, then the main fault option group (a capital letter) is listed.

#### Trace

The trace table provides a list of individual fault traces, the faults and main faults they have been assigned to, their location and activity certainty, and the attributes that have been considered to determine these. The traces are provided in the order of the faultName and traceNumber (see below). A trace is the lowest level of observation and is based on primary observations.

**traceKey** (Integer) – a unique identifier for each trace, presented in increasing integer order.

**traceName** (String) – a name assigned to each trace to help identify it. A combination of geographical and political locations as well as natural and man-made features has been used.

***faultName*** (String) – see explanation under Fault table. Each trace is assigned to a fault.

***mainFaultName*** (String) – obtained from fault name, see MainFault table. Each trace is assigned to a fault and each fault is assigned to a main fault.

**traceNumber** (Integer) – each trace is provided with a trace number showing its position along a fault. If there is only one trace for a fault, then the trace number will be 1. Where a fault has been interpreted as comprising multiple traces, the traces are ordered sequentially from one end to the other (for the central Apennines database this is generally ordered from northwest to southeast). Note there are some examples of faults where not every trace is considered to be part of the main fault, these are still assigned a number, but note the fault may not simply be the individual traces amalgamated together. Traces across strike from the main fault line are assigned a number in the hundreds in order to differentiate them from the main fault line. Traces with the same hundred number are located along strike from each other.

***faultNumber*** (String) – obtained from fault name, see Fault table. Identifies position of fault within main fault.

***traceLocationScale*** (Integer) – see TraceLocation table. This provides a gauge on how certain the location of the trace is. The score is derived from the observations in the Trace Table.

***traceActivityScale*** (Integer) – see TraceActivity table. This provides a gauge on how certain the activity of the trace is during the Late Pleistocene-Holocene. The score is derived from the observations in the Trace Table.

**slipSense** (String) – the type of slip experienced in the Late Pleistocene – Holocene along the fault – i.e. normal, strike or reverse

**bedrockFaultScarpGroundTruthed** (Integer) – a significant proportion of the trace has a bedrock fault scarp exposed, or bedrock fault scarps are exposed at regular intervals along the trace, these have been checked directly (on site). This field is displayed as “1” if the observation has been made and “0” or NULL if not.

**sharpQuaternaryTectonicScarpGroundTruthed** (Integer) – a significant proportion of the trace has a sharp Quaternary tectonic scarp, or scarps are exposed at regular intervals along the trace, these have been checked directly (on site). This field is displayed as “1” if the observation has been made and “0” or NULL if not.

**bedrockFaultScarpIdentifiedThroughRemoteSensingOrSatelliteImagery** (Integer) – a significant proportion of the trace has a bedrock fault scarp exposed, or bedrock fault scarps are exposed at regular intervals along the trace, these have been checked through remote techniques, but not necessarily *in situ*. This field is displayed as “1” if the observation has been made and “0” or NULL if not.

**sharpQuaternaryTectonicScarpIdentifiedThroughRemoteSensingOrSatelliteImagery** (Integer) – a significant proportion of the trace has a sharp Quaternary tectonic scarp, or scarps are exposed at regular intervals along the trace, these have been checked through remote techniques, but not necessarily *in situ*. This field is displayed as “1” if the observation has been made and “0” or NULL if not.

**changeInSlope** (Integer) – there is a change of slope identified along the trace. This can have been identified *in situ* or through remote techniques such as using a DEM. This field is displayed as “1” if the observation has been made and “0” or NULL if not.

**incisedDrainage** (Integer) – incised drainage has been identified along the trace either *in situ* or via remote techniques such as satellite imagery or using a DEM. This field is displayed as “1” if the observation has been made and “0” or NULL if not.

**changeInSlopeAndIncisedDrainageAndAlongFaultGroundTruthedScarpOrPrimarySurfaceRuptures** (Integer) – a change in slope and incised drainage have been identified along the trace, and on other traces belonging to the same fault (only including along-strike traces, i.e. only those with the same hundred number in the traceNumber), that a ground-truthed scarp (bedrock or other Quaternary) or primary earthquake surface ruptures have been identified. This field is displayed as “1” if the observation has been made and “0” or NULL if not.

**geophysicalData** (Integer) – data from geophysical equipment such as ground penetrating radar or seismic reflection profiling has been used to locate the fault trace at particular site(s). This field is displayed as “1” if the observation has been made and “0” or NULL if not.

**realTimePrimaryEarthquakeSurfaceRupturesDirectlyObservedInField** (Integer) – primary earthquake ruptures have been identified through direct observation (e.g. during a field campaign) within good time following an event. This field is displayed as “1” if the observation has been made and “0” or NULL if not.

**earthquakeDisplacementFromInSAROrSimilar** (Integer) – primary earthquake ruptures have been identified through remote sensing such as InSAR within good time following an event. This field is displayed as “1” if the observation has been made and “0” or NULL if not.

**palaeoseismicTrench** (Integer) – tectonic offsets have been identified in at least one trench site along the trace, with some form of dating constraining the offset to within the Late Pleistocene – Holocene. This field is displayed as “1” if the observation has been made and “0” or NULL if not.

**cosmogenicDating** (Integer) – cosmogenic isotope exposure dating has identified tectonic offset at least one site along the trace with the modelled data constraining offsets to within the Late Pleistocene - Holocene. This field is displayed as “1” if the observation has been made and “0” or NULL if not.

**offsetLatePleistoceneHoloceneDepositsMesauredThroughTopographicOffsets** (Integer) – tectonic offsets have been identified, the ages have been constrained through regional dating such as dating of a palaeosurface. This field is displayed as “1” if the observation has been made and “0” or NULL if not.

**datedOffsetAlongFaultWithLocalOrRegionalDating** (Integer) – along other traces belonging to the same fault (only including along-strike traces, i.e.only those with the same hundred number in the traceNumber), tectonic offsets have been identified; these will have either local dating or dating constrained through regional markers. This field is displayed as “1” if the observation has been made and “0” or NULL if not.

#### TraceLocation

The trace location table contains the scale used to describe how certain the location of the trace is.

**locationScale** (Integer) – an integer value (1–4, 1 = highest) indicating the certainty in the trace location.

**locationDescription** (String) – explanation of the trace certainty scale.

**locationDisplay** (Number) – how the trace is displayed in the map, i.e. the line thickness (in pixels) with a thicker line representing higher location certainty

#### TraceActivity

The trace activity table contains the scale used to describe the level of certainty that there has been tectonic activity along the trace since the Late Pleistocene - Holocene

**activityScale** (Integer) – an integer value (1–4, 1 = highest) indicating the certainty in the trace activity.

**activityDescription** (String) – explanation of the trace activity scale.

**activityDisplay** (String) – how the trace is displayed in both the GIS map and GoogleEarth, i.e. the line colour with red indicating the strongest evidence for Late Pleistocene-Holocene activity and grey being the least.

#### SlipRate

The slip rate table provides slip rate and throw rate measurements and their locations along the traces. Slip-rates are associated with individual traces and grouped by fault.

**slipRateKey** (Integer) – unique identifier for each slip rate row

***traceName*** (String) – see Trace table

***faultName*** (String) – obtained from trace name, see Fault table. Each trace is assigned to a fault.

***mainFaultName*** (String) – this is a dynamic field obtained from fault name, see MainFault table. Each trace is assigned to a fault and each fault is assigned to a main fault.

***traceNumber*** (Integer) – obtained from the trace name, see Trace table.

***faultNumber*** (Integer) – this is a dynamic field obtained from the fault name, see Fault table.

**slipRatePreferredMmYr** (Number) – the “preferred” slip-rate expressed in mm yr^−1^. This is the slip-rate calculated using the author’s most likely slip and most likely time window over which the slip was accumulated. Note the “preferred” value is interpreted to be the mean value, unless an explicit “preferred” value is reported. Calculated as slipPreferredM/timeWindowPreferred, unless a direct measurement for slip-rate itself is provided. Note the slip-rate has to be defined at a specific location.

**slipRateMinimumMmYr** (Number) – the “minimum” slip-rate expressed in mm yr^−1^. This is the slip-rate calculated using the author’s minimum slip (ie “preferred” or “mean” slip minus the error) and maximum time window over which the slip was accumulated (i.e. the time window + error in the time window; this is calculated as the time between the oldest older age and youngest younger age deemed possible). Calculated as slipMinimumM/timeWindowMaximum, unless a direct measurement for the minimum slip-rate itself is provided. Note the slip-rate has to be defined at a specific location.

**slipRateMaximumMmYr** (Number) – the “maximum” slip-rate expressed in mm yr^−1^. This is the slip-rate calculated using the author’s maximum slip (ie “preferred” or “mean” slip plus the error) and minimum time window over which the slip was accumulated (i.e. the time window - error in the time window; this is calculated as the time between the youngest older age and oldest younger age deemed possible). Calculated as slipMaximumM/timeWindowMinimum, unless a direct measurement for the maximum slip-rate itself is provided. Note the slip-rate has to be defined at a specific location.

**throwRatePreferredMmYr** (Number) - vertical component of slipRatePreferredMmYr expressed in mm yr^−1^

**throwRateMinimumMmYr** (Number) - vertical component of slipRateMinimumMmYr expressed in mm yr^−1^

**throwRateMaximumMmYr** (Number) - vertical component of slipRateMaximumMmYr expressed in mm yr^−1^

**xCoordinateUTM** (Number) - the x UTM coordinate of the site where the measurement was taken. See coordinateSystemKey that informs whether this was the original coordinate system used to determine the location or whether it has been derived.

**yCoordinateUTM** (Number) – the y UTM coordinate of the site where the measurement was taken. See coordinateSystemKey that informs whether this was the original coordinate system used to determine the location or whether it has been derived.

**gridUTM** (String) – the UTM grid box of the UTM coordinates.

**xCoordinateWGS84** (Number) – the longitude coordinate of the site where the measurement was taken in decimal degrees in the WGS 84 system. See coordinateSystemKey that informs whether this was the original coordinate system used to determine the location or whether it has been derived.

**yCoordinateWGS84** (Number) – the latitude coordinate of the site where the measurement was taken in decimal degrees in the WGS 84 system. See coordinateSystemKey that informs whether this was the original coordinate system used to determine the location or whether it has been derived.

**coordinateErrorM** (Number) – the estimated error, measured in metres, of the location (ie in the x and y coordinate)

***coordinateSystemKey*** (Number) – specifies the original coordinate system used to measure the location – see Coordinate System Table.

***techniqueKeyThrowSlip*** (Integer) – the method used for measuring the throw or slip. See Technique Table.

***referenceKeyThrowSlip*** (String) – the publication from which the throw or slip measurement has been taken. See Reference Table.

***techniqueKeyDate*** (Integer) – the method used for measuring the time period over which the throw and/or slip occurred. See Technique Table.

***referenceKeyDate*** (String) – the publication from which the time period has been taken. See Reference Table.

**slipPreferredM** (Number) – the “preferred” slip expressed in metres. Note the “preferred” value is interpreted to be the mean value, unless an explicit “preferred” value is reported. See originalMeasurement to determine whether the slip was measured directly, or calculated from the throw measurement. The younger and older age between which the slip occurred is shown in the Age columns.

**slipMinimumM** (Number) – the minimum slip expressed in metres. See originalMeasurement to determine whether the slip was measured directly, or calculated from the throw measurement.

**slipMaximumM** (Number) – the maximum slip expressed in metres. See originalMeasurement to determine whether the slip was measured directly, or calculated from the throw measurement.

**throwPreferredM** (Number) – the “preferred” throw expressed in metres. Note the “preferred” value is interpreted to be the mean value, unless an explicit “preferred” value is reported. See originalMeasurement to determine whether the throw was measured directly, or calculated from the slip measurement. The younger and older age between which the throw occurred is shown in the Age columns.

**throwMinimumM** (Number) – the minimum throw expressed in metres. See originalMeasurement to determine whether the throw was measured directly, or calculated from the slip measurement.

**throwMaximumM** (Number) – the maximum throw expressed in metres. See originalMeasurement to determine whether the throw was measured directly, or calculated from the slip measurement.

**youngerAgePreferredKA** (Number) – the “preferred” age for the younger age of the time window over which the slip or throw was measured, measured in thousands of years before present. Note the “preferred” value is interpreted to be the mean value, unless an explicit “preferred” value is reported.

**youngerAgeMinimumKA** (Number) – the youngest age for the younger age of the time window over which the slip or throw was measured, measured in thousands of years before present.

**youngerAgeMaximumKA** (Number) – the oldest age for the younger age of the time window over which the slip or throw was measured, measured in thousands of years before present.

**olderAgePreferredKA** (Number) – the “preferred” age for the older age of the time window over which the slip or throw was measured, measured in thousands of years before present. Note the “preferred” value is interpreted to be the mean value, unless an explicit “preferred” value is reported.

**olderAgeMinimumKA** (Number) – the youngest age for the older age of the time window over which the slip or throw was measured, measured in thousands of years before present.

**olderAgeMaximumKA** (Number) – the oldest age for the older age of the time window over which the slip or throw was measured, measured in thousands of years before present.

**timeWindowPreferredKA** (Number) – the “preferred” time window over which the slip or throw was measured, measured in thousands of years. Calculated as olderAgePreferredKA – youngerAgePreferredKA.

**timeWindowMinimumKA** (Number) – the minimum time window over which the slip or throw was measured, measured in thousands of years. Calculated as olderAgeMinimumKA – youngerAgeMaximumKA.

**timeWindowMaximumKA** (Number) – the maximum time window over which the slip or throw was measured, measured in thousands of years. Calculated as olderAgeMaximumKA – youngerAgeMinimumKA.

**originalMeasurement** (String) – states whether the original measurement stated in the reference was a throw or slip.

**throwSlipConversionMethod** (Number) – refers to the technique used to convert from throw to slip or slip to throw. See technique table.

#### LocalGeometryKinematics

The local geometry and kinematics table provides local surface slip vector, strike and dip measurements and the locations at which they were measured. Note only direct point measurements are included. Geometry measurements are associated with individual traces and grouped by fault. There are no data included regarding the depth of the fault or how surface measurements may project to depth as we do not consider that currently there are sufficient constraints for this to be included; a modeller will need to add their own assumptions.

**geometryKey** (Integer) – unique identifier for each row

***traceName*** (String) – see Trace table

***faultName*** (String) – obtained from trace name, see Fault table. Each trace is assigned to a fault.

***mainFaultName*** (String) – this is a dynamic field obtained from fault name, see MainFault table. Each trace is assigned to a fault and each fault is assigned to a main fault.

***traceNumber*** (Integer) – obtained from the trace name, see Trace table.

***faultNumber*** (Integer) – this is a dynamic field obtained from the fault name, see Fault table.

**strike** (Integer) – strike measurement at the location specified

**surfaceDip** (Integer) – dip measurement at the location specified, measured at the surface

**slipVectorAzimuth** (Integer) – slip vector azimuth at the location specified, measured at the surface

**slipVectorPlunge** (Integer) – slip vector plunge at the location specified, measured at the surface

**xCoordinateUTM** (Number) – the x UTM coordinate of the site where the measurement was taken. See coordinateSystemKey that informs whether this was the original coordinate system used to determine the location or whether it has been derived.

**yCoordinateUTM** (Number) – the y UTM coordinate of the site where the measurement was taken. See coordinateSystemKey that informs whether this was the original coordinate system used to determine the location or whether it has been derived.

**gridUTM** (String) – the UTM grid box of the UTM coordinates.

**xCoordinateWGS84** (Number) – the longitude coordinate of the site where the measurement was taken in decimal degrees in the WGS 84 system. See coordinateSystemKey that informs whether this was the original coordinate system used to determine the location or whether it has been derived.

**yCoordinateWGS84** (Number) – the latitude coordinate of the site where the measurement was taken in decimal degrees in the WGS 84 system. See coordinateSystemKey that informs whether this was the original coordinate system used to determine the location or whether it has been derived.

**coordinateErrorM** (Number) – the estimated error, measured in metres, of the location (ie in the x and y coordinate)

***coordinateSystemKey*** (Number) – specifies the original coordinate system used to measure the location – see Coordinate System table.

***techniqueKeyGeometry*** (Integer) – the method used for measuring the slip vector or surface fault geometry. See Technique Table.

***referenceKeyGeometry*** (String) – the publication from which the measurements have been taken. See Reference Table.

#### Earthquakes

The earthquakes table provides a summary of references to instrumental, historical and palaeoearthquakes along the traces. Note the list is not exhaustive.

**earthquakeKey** (Integer) – unique identifier for each row

***traceName*** (String) – see Trace table

***faultName*** (String) – obtained from trace name, see Fault table. Each trace is assigned to a fault.

***mainFaultName*** (String) – this is a dynamic field obtained from fault name, see MainFault table. Each trace is assigned to a fault and each fault is assigned to a main fault.

***tehniqueKeyEarthquake*** (Integer) – the method used for determining contemporary or palaeoearthquakes along the trace. See Technique table.

***referenceKeyEarthquake*** (String) – the publication from which the data have been taken. See Reference table.

#### CoordinateSystem

States the coordinate system that relates to the coordinate system key, which is used in point data tables to identify the original coordinate system used for locating data collection sites and the technique for converting between the original coordinate system of the measurement and those displayed in the data tables.

**coordinateSystemKey** (Integer)– unique identifier for each coordinate system of the original location reported in the reference

**originalCoordinateSystem** (String) – the coordinate system corresponding to the coordinateSystemKey

**finalCoordinateSystem** (String) – the coordinate system that the original coordinates were converted to

**coordinateConversionTechnique** (String) – the method used for converting the coordinates

#### Technique

States the technique used for the measurement

**techniqueKey** (Integer) – unique identifier for each technique

**techniqueDescription** (String) – the technique used for taking the measurement

#### Reference

A table of references cited in the database.

**referenceKey** (String) – unique identifier for each reference in the table, it has the format of the author name(s) followed by year of publication.

**referenceShortName** (String) – reference abbreviation in Harvard citation format.

**articleTitle** (String) – title of reference

**authors** (String) – full list of authors

**year** (Integer) – year of publication

**journal** (String) – name of journal

**doi** (String) – doi of publication

**volume** (Integer) – volume of publication

**issue** (Integer) – issue number, this field will be a “NULL” if there is no issue number

**firstPage** (Integer) – first page of article, this field will be a “NULL” if there are no page numbers

**lastPage** (Integer) – first page of article, this field will be a “NULL” if there are no page numbers

## Technical Validation

In the central Apennines database, we have defined 43 or 44 individual main faults (depending on main fault selection chosen, selection is made from a choice of 49 main faults, but only 43 or 44 are independent) made up of 87 faults which are discretised into 312 traces. The number of traces per fault varies from 1 up to 16. The number of faults per main fault varies from 1 up to 5. The number of traces per main fault varies from 1 up to 51.

Table 3 provides the percentage of traces, faults and main faults with the different levels of location certainty and activity. The activity level for each main fault is assigned as the highest from among its constituent faults and fault activity is assigned as the highest from among its constituent traces, so that all evidence of activity is included in defining the activity of the fault. Of the 43/44 main faults included within any set of main fault selections, 38/39 have confirmed Holocene – Late Pleistocene activity (Activity Level 1 or 2) (44 of the 49 possible main faults listed). The remaining 5 main faults do not have confirmed activity in the Late Pleistocene-Holocene; they are included to encourage further study and so their potential activity can be included in hazard models. As the assigned location certainty for each main fault is the lowest level from among its constituent faults (and likewise for faults from among its traces), a main fault and fault will have low location certainty even if parts of them are well-constrained if there are traces along a fault which are less-well constrained, as is common towards the surface tips.

**Table 3:** Percentage of main faults, faults and traces with different levels of activity and location certainty. The range of values for the main faults arises from the different combinations of main fault selection options.

LEVEL	Main Faults Activity	Faults Activity	Traces Activity	Main Faults Location Certainty	Faults Location Certainty	Traces Location Certainty
1	77%	56%	36%	7%	9%	40%
2	11–12%	14%	12%	11–14%	20%	25%
3	0%	0%	32%	19–20%	20%	10%
4	11–12%	30%	20%	59–63%	52%	25%

**Fig. 4:**
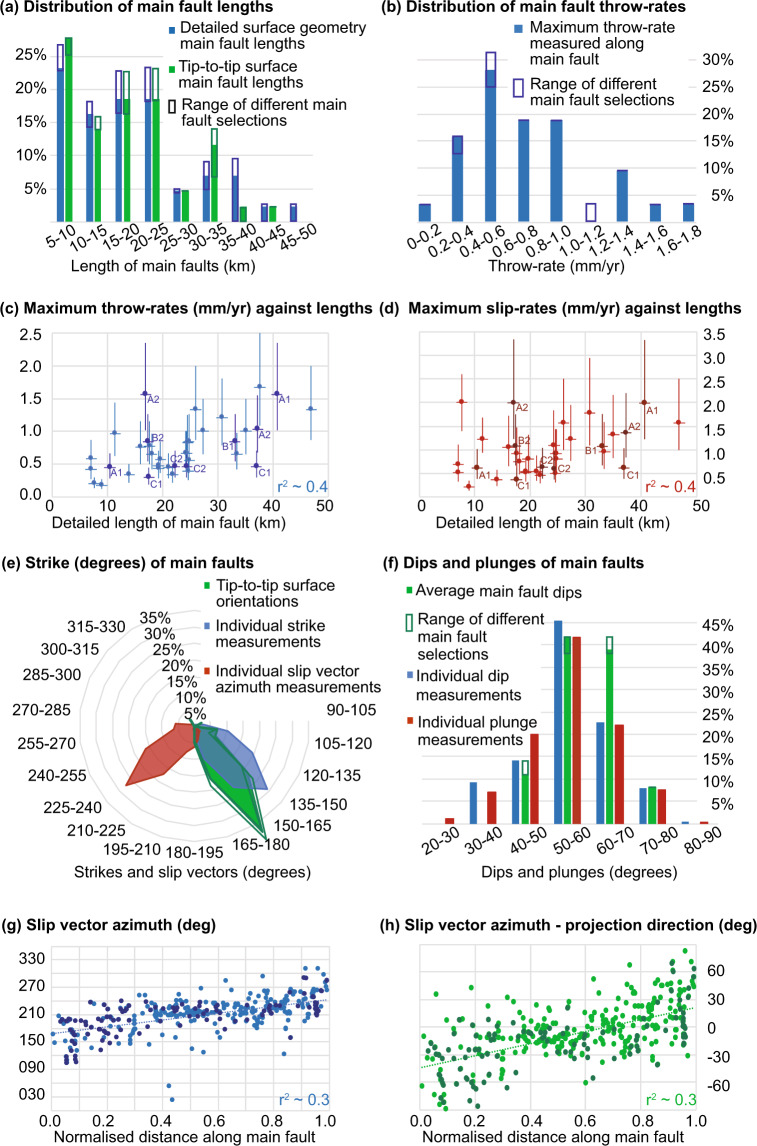
Summary of individual measurements and main fault-averaged values within the database. Note not all main faults have data. The figures show the range of values obtained from different main fault option choices, either with solid bars showing main fault option choices A1, B1 and C1, with the open boxes in darker colours showing the range of values obtained from different combinations of main fault option choices, or for the scatter graphs explicitly labelling the points with alternate values dependent on main fault option choices. (**a**) Main fault lengths, shown for both the detailed surface paths and linear tip-to-tip lengths. (**b**) Maximum throw-rates along individual main faults, the percentages shown are of the main faults with data. (**c**) Maximum throw-rates along individual main faults versus detailed main fault length (note estimated values are excluded). (**d**) Maximum slip-rates along individual main faults versus detailed main fault length (note estimated values are excluded). (**e**) Average surface tip-to-tip orientations of main faults, individual strike measurements, and individual slip vector azimuth measurements. (**f**) Weighted average dips per main fault, all measured dips and plunges. (**g**) All measured slip vector azimuths versus normalised distances along main faults. (**h**) All measured slip vector azimuths minus the slip projection direction (i.e. the difference between the slip vector and the dip-slip slip vector azimuth of the fault assuming the dip-slip value is perpendicular to the tip-to-tip strike of the main fault) for the main fault versus normalised distances along main faults.

Main faults in the region vary in length from 6.0 km up to 44.4 km or 6.5 km up to 46.9 km depending whether length is measured as a tip-to-tip length, or if the detailed surface geometry is considered (Fig. 4a). Faults vary in length from 1.2 km up to 44.4 km or 1.3 km up to 46.9 km for tip-to-tip and detailed surface geometry respectively. Errors in the main fault and fault lengths are hard to define as surface fault tips and the lateral extent of total displacement are difficult to locate with precision, especially for faults with low slip-rates and small total offsets relative to the thickness of geological units.

Table 4 provides a summary of the proportion of traces, faults and main faults that have at least one point measurement of (a) throw and/or slip-rate, (b) strike, (c) surface dip, (d) slip vector azimuth, and (e) slip vector plunge.

**Table 4:** Percentage of traces, faults and main faults that contain at least one point measurement of various parameters within the database. The range of values for the main faults arises from the different combinations of main fault selection options.

	Main Faults	Faults	Traces
Throw-rate and/or slip-rate	70–72%	46%	20%
Strike	70–72%	45%	18%
Surface dip	70–72%	45%	18%
Slip vector azimuth	73–74%	46%	25%
Slip vector plunge	73–74%	46%	24%

The database contains 149 point measurements of throw-rate and/or slip-rate. These are distributed among 61 traces along 40 faults and 31 main faults. Therefore, 20% of traces, 46% of faults, and 70–72% of main faults have the slip-rate and/or throw-rate constrained in at least one location. Measured preferred throw-rates and slip-rates at individual sites vary up to 1.7mmyr^−1^and 2.0mmyr^−1^ respectively (Fig. 4b) and the mean measured maximum throw-rate is 0.7 mmyr^−1^ on both main faults and faults. There is a modest correlation (R^2^~0.4 depending on mainFault selections) between the maximum measured throw-rates and slip-rates and the lengths of the main faults (Fig. 4c,d). Note there are some further constraints on slip-rates from the literature, but the database only contains data that meet the criteria for inclusion.

The database contains 468 point measurements of strike. These are distributed among 57 traces along 39 faults and 31 main faults. Therefore, 18% of traces, 45% of faults and 70–72% of main faults have local strike constrained in at least one location. The modal group of individual strike measurements is 120° -135°, while for the surface tip-to-tip orientations it is 135°-150° for faults and main faults. 81%, 85% and 80% of the individual strike measurements, tip-to-tip surface main fault orientations and tip-to-tip surface fault orientations respectively lie between 105° and 165°, while 93%, 96% and 90% respectively lie between 90° and 180°, and hence can be described as having a SE strike (Fig. 4e).

The database contains 470 point measurements of surface dip. These are distributed among 56 traces along 39 faults and 31 main faults. Therefore, 18% of traces, 45% faults and 70–72% of main faults have the dip constrained in at least one location. The mean and median of the individual surface dip measurements are 56°, with a standard deviation of 10° and all measurements having a range between 31° and 85°. The weighted average mean dip for main faults in the region is 59°, with a standard deviation of 7°, the median is 60° and the range is 43°–74° (Fig. 4f).

The database contains 290 locations with the slip vector azimuth constrained. These are distributed among 77 traces along 40 faults and 32 main faults. Therefore, 25% of traces, 46% of faults and 73–74% of main faults have the local slip vector azimuth constrained in at least one location. The mean of the measured slip vector azimuths is 211°, with a standard deviation of 37° (Fig. 4e); the measured slip vector azimuths are consistent with dip slip faults with converging patterns of slip (Fig. 4g,h), although only a weak correlation (R^2^~0.3) is shown (note the correlation for Fig. 4g increases to 0.4 if only southwest dipping main faults are included).

The database contains 254 locations with the slip vector plunge constrained. These are distributed among 76 traces along 40 faults and 32 main faults. Therefore, 24% of traces, 46% of faults and 73-74% main faults have the local slip vector plunge constrained in at least one location. Individual measurements range between 23° and 82°, with a median value of 56°, mean value of 55° and standard deviation of 10° (Fig. 4f).

## Usage Notes

The data can be extracted for use in fault-based hazard assessments, in particular the modeller has access to the primary data so has the potential to include detailed uncertainty analyses. The modeller can decide which level of mapping (main faults, faults, traces) to incorporate within their hazard model (Fig. 1a). At the main fault level, we have provided some alternate options where there are different interpretations of how to connect faults. The modeller will need to decide how they allow earthquakes to rupture, for example whether multi-fault ruptures are possible and if so, what are the rules governing such ruptures (Fig. 1b). Depending on model capability, the modeller will have to determine how to extrapolate slip-rate measurements along the main fault or and/or fault (Fig. 1c). How the data is used may be dependent on the scale of the model being created – for example whether local surface rupture and high precision are required or if it is a lower resolution hazard map, and whether the model is aiming to provide information on annual probabilities or investigating a worst credible event.

Examples of tools for fault-based SHA include SHERIFS^101^ and FiSH codes^27^. To aid readers use the data for both these codes, we provide tools for extracting the data into the appropriate formats. (For an example of using the data for SHA input see^37^).

The database structure can be used as a template for data reporting within the central Apennines and with possible adaptations for other regions. We suggest that data from field investigations should be reported such that the data are useful for and can be easily included into SHA calculations both within the capabilities of current models and in the future. Therefore, we advocate that all authors reporting fault data - in particular slip-rate and fault geometry – should do so in a manner that would be fit for incorporation in a database structure that we recommend herein. We suggest fault traces should be displayed with accompanying notes on the location and activity certainty and the evidence for these (in auxiliary files if required). We propose that all slip-rate and fault geometry measurements should be reported with their spot location details including precise coordinates, the primary data collected, the methods used, and the uncertainties in the data. Slip-rates should provide time periods over which they are estimated including the techniques and errors used to infer these. Such transparency will allow better incorporation of primary data uncertainties and the propagation of these uncertainties within hazard modelling. In turn, this can lead to better understanding of the uncertainty in risk calculations used in catastrophe modelling and risk assessments used by governments, civil protection and the insurance industry to manage and reduce risk to residents, industry and society as a whole.

The database should also be useful to those planning field investigations requiring fault data for purposes beyond SHA.

It is our intention to update the database with new information and expand the geographical area covered. Note new releases are unlikely to be more frequent than annually. The next release will likely include (1) additional areas to the northwest and southeast of the current area covered; (2) more details within the Earthquakes tables regarding individual events; and (3) more data input for current traces if received from contributors. Contributors will be acknowledged. There will be consideration as to whether to include slip-rate data that has not been provided with a specific grid reference, but can constrain the slip-rate along a fault.

## Data Availability

A MATLAB script (data2FiSH.m) for extraction of the data from the database in a ready-to-use format for the FiSH code and an accompanying readme text file (README_script_data2FISH.txt) with instructions for its use. These are contained within the “Data2FiSH” folder along with 2 supporting MATLAB scripts: “utm2deg.m” and “deg2utm.m”.
